# Impact of different post-polymerization protocols on fracture load and two-body wear of 3D-printed fixed dental prostheses

**DOI:** 10.1007/s00784-026-07232-9

**Published:** 2026-10-22

**Authors:** Sophie Gekle, Bogna Stawarczyk, Felicitas Mayinger, Ursula Kreitmair, Moritz Hoffmann, John Meinen, Marcel Reymus

**Affiliations:** 1 Department of Prosthetic Dentistry, University Hospital, LMU Munich, Goethestrasse 70, Munich, 80336 Germany; 2 Biomaterials and Technology, Department Research, University Center for Dental Medicine Basel UZB, University of Basel, Basel, Switzerland; 3 Department of Reconstructive Dentistry, University Center for Dental Medicine Basel UZB, University of Basel, Basel, Switzerland; 4 NK Optik GmbH, Baierbrunn, Germany; 5 Department of Conservative Dentistry, Periodontology and Digital Dentistry, University Hospital, LMU Munich, Munich, Germany

**Keywords:** 3D printing, Digital light processing, Post-polymerization, Fracture load, Two-body wear, Dental prostheses

## Abstract

**Objectives:**

To evaluate five post-polymerization protocols (385, 400 and 460 nm LEDs; one- and two-capacitor flashlights) and aging on fracture load (FL) and two-body wear (TBW) of three 3D-printed resins for fixed dental prostheses (FDPs).

**Materials and methods:**

360 single-unit FDPs for mandibular molars were DLP-printed from two permanent resins (Varseo Smile CrownPlus [VAR], Crowntec [CRO]) and one temporary resin (Freeprint temp [FRE]). Half was artificially aged (480,000 mastication cycles, thermocycling 5/55°C). FL was measured in a universal testing machine, TBW by laser scanning. Data were analysed with three-way ANOVA, Kruskal–Wallis and post-hoc tests (α = 0.05).

**Results:**

The protocol (*p* < 0.001) and the material (*p* < 0.001) significantly affected FL, whereas aging showed no significant main effect (*p* = 0.100); the three-way interaction was significant (*p* = 0.009). Initially, 460 nm yielded the highest FL for VAR (1505 ± 251 N) and FRE (1910 ± 237 N), the two-capacitor flashlight for CRO (1783 ± 333 N). Aging increased FL significantly in two and decreased it in three of 15 combinations. TBW (− 0.16 to − 0.35 mm) did not differ between protocols or materials.

**Conclusions:**

The protocol effect on FL depended on the material and the aging level. Aging had no overall effect but increased FL in two combinations, possibly through continued polymerization. Protocol selection should consider the material.

**Clinical relevance:**

Mean fracture loads exceeded reported maximum molar bite forces, although individual specimens failed below that range. Two-capacitor flashlight post-curing gave the most consistent performance for the materials tested.

## Introduction

Additive manufacturing, also referred to as 3D printing, provides a precise and efficient method for producing both provisional and permanent fixed dental prostheses (FDPs). Compared with conventional fabrication methods, this approach reduces fabrication time, material loss, and material wear, thereby offering a cost-effective alternative [1]. In dentistry, the most commonly used 3D-printing technique is vat photopolymerization (VAT), specifically stereolithography (SLA) and digital light processing (DLP). These techniques enable the production of restorations with flexural strengths ranging from 60 to 90 MPa and fracture resistance values of 1000–1200 N, which are comparable to those of conventionally fabricated provisional restorations [1]. However, 3D-printed FDPs are still subject to further development, as they do not yet match the performance of traditionally manufactured restorations in all studies [2, 3]. In direct comparison, higher biaxial flexural strength has been demonstrated by CAD/CAM-milled permanent resins than by 3D-printed materials. Subtractive manufactured composite resin crowns have also exhibited superior dimensional stability and lower wear rates [4, 5]. However, recent studies suggest that properly processed 3D-printed restorations can achieve fracture loads (FL) exceeding 1000 N, thereby meeting the minimum clinical requirements for posterior restorations [6].

]Particularly for permanent applications, stricter demands are required, as permanent restorations must withstand long-term occlusal forces. Mechanical properties such as flexural strength and surface hardness must meet established requirements to remain competitive with other fabrication methods [7]. The incorporation of various inorganic fillers is intended to enhance mechanical properties [8]; consequently, resins designed for permanent use contain a higher filler content compared to those intended for provisional applications, achieving higher mechanical properties [9]. Filler-matrix interactions are of considerable significance in determining material properties. Both filler type and filler content influence these properties, particularly fracture resistance and wear behaviour [7]. The incorporation of specific fillers, including silica-based particles, zirconia nanoparticles, silica glass nanoparticles, and nanoparticle clusters, has been shown to improve flexural strength and biocompatibility through reinforcement mechanisms [10]. Filler distribution and size also affect fracture behaviour; notably, uneven filler distribution has been found to have a more positive effect on FL than uniform distribution [11].

Post-polymerization has been identified as a critical step in the fabrication workflow of 3D-printed dental restorations, as it directly influences the degree of conversion and, consequently, the mechanical properties of the final restoration [12]. Various light sources and protocols are utilized for post-polymerization, including LED-based devices with specific wavelengths and stroboscopic flashlight systems. However, the optimal parameters for post-polymerization protocols continue to be the subject of ongoing investigation, with limited comparative evidence regarding the effects of different light sources on the long-term mechanical performance of the printed restorations. Recent systematic reviews have indicated that post-curing times of 60–120 min, which significantly exceed manufacturer recommendations of 15–30 min, yield optimal mechanical properties; higher temperatures (> 60 °C) have been shown to accelerate monomer diffusion and crosslinking [13]. The degree of conversion is critically dependent on the alignment between the absorption spectrum of the containing photoinitiatorsystem and the curing light wavelength: diphenyl(2,4,6-trimethylbenzoyl)phosphine oxide (TPO) exhibits peak absorption at 365–385 nm, whereas bis-acylphosphine oxide (BAPO) shows maximum absorption at approximately 405 nm [14]. Mismatches in wavelength have been found to result in incomplete polymerization, leading to residual monomers, reduced crosslink density, and inferior mechanical properties [15]. Additionally, a printing orientation of 0° has been associated with the highest flexural strength, with time-dependent improvements in mechanical properties observed up to 120 min of post-curing; this has been corroborated by SEM analysis, which revealed increasingly ductile fracture patterns [16].

The objective of this investigation was to evaluate the impact of two different light sources, LED lights at three wavelengths (385 nm, 400 nm, 460 nm) and a stroboscopic flashlight, as well as the effect of artificial aging on the mechanical properties of three resin materials: two intended for permanent use and one for temporary application. The null hypotheses tested were that: (a) the choice of post-polymerization protocol would not significantly affect FL or two-body wear (TBW), and (b) artificial aging would not significantly influence FL or TBW.

## Materials and methods

### Study design and ethical considerations

This in vitro study did not involve human subjects, tissues, or data; therefore, ethical approval was not required.

### Sample size

A sensitivity analysis of the achievable resolution was performed instead of an a priori power calculation. The sample size of *n* = 12 specimens per group was adopted from a previous investigation on the same materials and post-polymerization protocols [12]; no a priori power analysis was performed. With *n* = 12 per group, α = 0.05 and a power of 0.80, a standardized between-group difference of d = 1.19 can be detected in a two-sided two-sample t-test. For two-body wear, the pooled standard deviation of vertical height loss was 0.160 mm, so the smallest detectable difference is 0.190 mm; this is of the same magnitude as the largest difference between subgroup means observed in the present study (0.191 mm). The two-body wear analysis is therefore not powered to exclude small differences between protocols or materials, and all two-body wear results are reported as exploratory.

### Specimen preparation

**Fig. 1:**
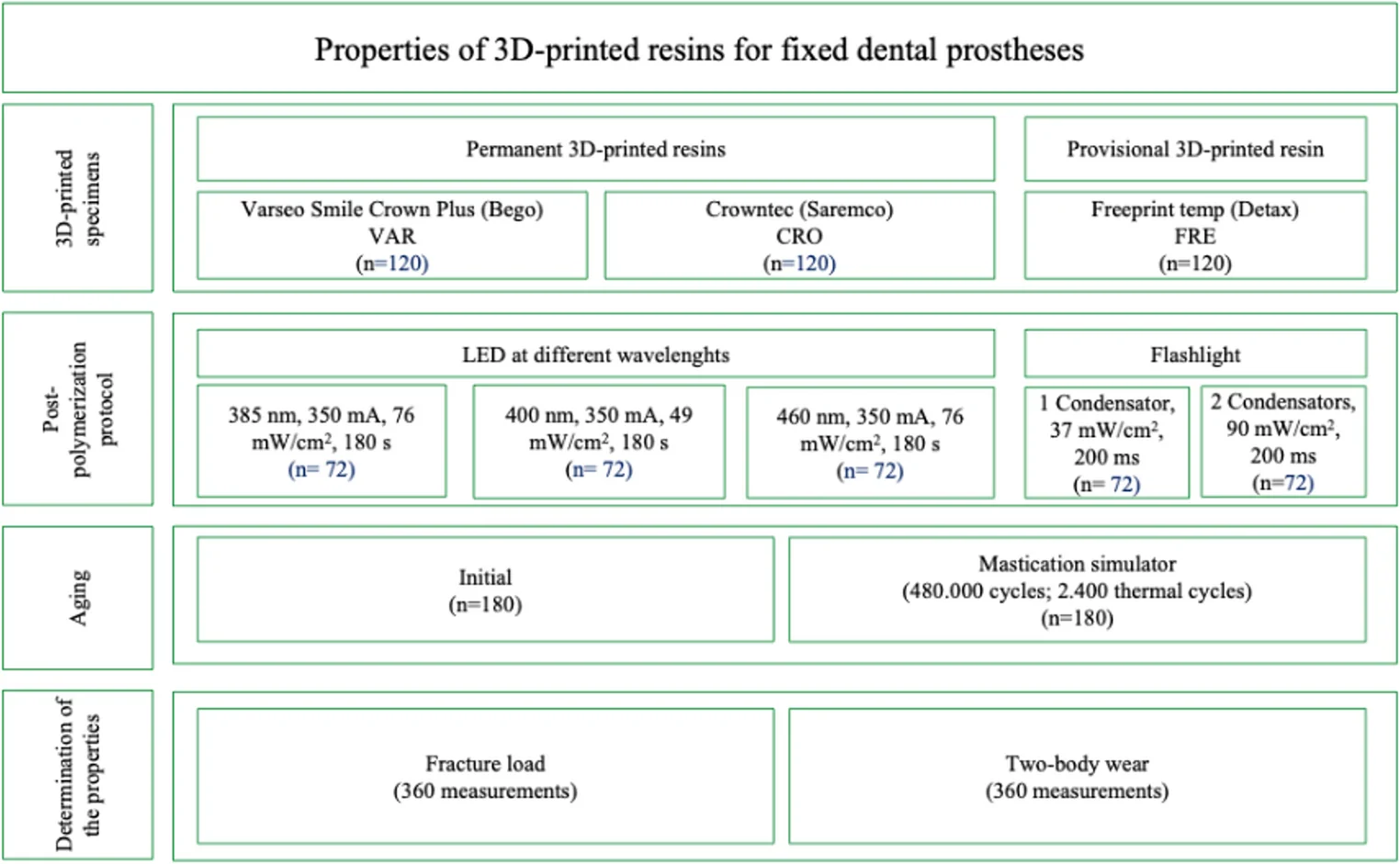
Study design

A total of 360 single-unit fixed dental prostheses (FDPs) for a mandibular first molar were designed using a master standard tessellation language (STL) file. Fabrication was carried out using two photopolymerizable resins intended for permanent use: VAR and CRO (Crowntec; Saremco Dental, Rebstein, Switzerland) as well as one resin for temporary use: FRE (Fig. 1; Table 1).

**Table 1:** Material, shade, LOT number, manufacturer, composition of materials used

Material	Abbreviation	Shade	LOT No.	Manufacturer	Composition
Varseo Smile Crown^Plus^	VAR	A1	601,374	BEGO Medical GmbH	esterification products of 4,4‘- isopropylidendiphenol, ethoxylated and 2-methylprop-2- enoic acid, silicon oxide, methylbenzoylformate, diphenyl (2,4,6-trimethylbenzoyl) phosphine oxide, inorganic fillers (30 to 50 wt%)
Crowntec	CRO	A1	F046	Saremco Dental AG	esterification products of 4,4`-isoprpoylidiphenol, ethoxylated and 2-methylprop-2- enoic acid, silanized dental glass, pyrogenic silica, initiators, inorganic fillers (30 to 50 wt%)
Freeprint temp	FRE	A1	260,704	Detax GmbH	methacrylate, phosphinoxide

The crowns were printed using a DLP printer (Asiga Max UV; Asiga Europe, Erfurt, Germany) at a layer thickness of 50 μm. Support structures were attached to the occlusal surface of the specimens, enabling a printing orientation of 0°. Following printing, the specimens underwent mechanical cleaning in a centrifuge (Multifuge 1 S-R; Heraeus, Hanau, Germany) for 4 min at 20 °C and 1500 rpm.

For post-polymerization, two experimental devices (NK Optik, Baierbrunn, Germany) were used, utilizing five distinct post-polymerization protocols (PP) (Table 2):

**Table 2:** Post-polymerization device, technology, wavelength, post-polymerization protocol and manufacturer

Post-polymerization device	Technology	Wavelength	Post-polymerization protocol	Manufacturer
Experimental post-polymerization device	Light-emitting diode	385, 400, 460 nm	1. 350 mA, 15 V, 385 nm, 180 s, 2. 350 mA, 15 V, 400 nm, 180 s 3. 350 mA, 15 V, 460 nm, 180 s	NK Optik GmbH (Baierbrunn, Germany)
Enhanced experimental Otoflash G171 device	Flashlight	280–700 nm, peaks at 400 nm and 500 nm	4. 1 capacitor, maximum intensity, 2000 flashes 5. 2 capacitors, maximum intensity, 2000 flashes	NK Optik GmbH (Baierbrunn, Germany)

- PP 1: LED light (350 mA, 15 V, 385 nm, 180 s).
- PP 2: LED light (350 mA, 15 V, 400 nm, 180 s).
- PP 3: LED light (350 mA, 15 V, 460 nm, 180 s).
- PP 4: Flashlight (one capacitor at maximum intensity for 2000 flashes, 280–700 nm, peaks at 400 nm and 500 nm).
- PP 5: Flashlight (two capacitors at maximum intensity for 2000 flashes, 280–700 nm, peaks at 400 nm and 500 nm).

During post-polymerization, only the intaglio surface of each specimen was oriented toward the light source. After post-polymerization, the support structures were removed with a scalpel, and the occlusal surface of each crown was polished using a goat-hair brush (Rundbürste, Ziegenhaar weiß; bredent medical, Senden, Germany) and polishing paste (Abraso Starglanz; bredent medical).

The specimens were then attached to alloy abutments simulating prepared mandibular first molars, with a height of 5.8 mm, a 360° chamfer preparation, and a taper of 6° (CoCr), using an autopolymerizing composite cement (els cem; Saremco Dental, Rebstein, Switzerland). Excess cement was removed, and the specimens were post-polymerized for 20 s on each side (Elipar DeepCure-L; 3 M, Seefeld, Germany). Finally, the specimens were stored in distilled water at 37 °C for 24 h (HeraCell 150; Kulzer, Hanau, Germany).

### Artificial aging

Half of the specimens in each subgroup (*n* = 180) were subjected to artificial aging. The specimens and their corresponding stainless-steel antagonists (X5CrNi 18 − 10; SD Mechatronik, Feldkirchen-Westerham, Germany) were mounted in a masticatory chewing simulator (CS-4.10; SD Mechatronik). Artificial aging was performed using 480,000 mastication cycles, with a vertical load of 50 N applied at a frequency of 1.3 Hz. Concurrently, a lateral movement of 0.7 mm, from the central fissure toward the buccal cusps, was introduced. During the mastication simulation, the specimens were also exposed to 2,400 thermal cycles in distilled water, alternating between temperatures of 5 °C and 55 °C, with each cycle lasting 60 s.

### Two-body wear measurement

The occlusal surfaces of the specimens were analysed using a laser scanner (LAS-20; SD Mechatronik) both before and after mastication simulation. The scanner was configured with a horizontal resolution of 40 μm and a vertical resolution of 0.8 μm, and the material type was set to “plastic matt.” To determine two-body wear, the generated scan data were imported into matching software (ZEISS INSPECT Optical 3D, 2024; Carl Zeiss AG, Oberkochen, Germany) and converted into a three-dimensional mesh structure. This process employed a step size of 0.0 mm, a maximum edge length of 0.15 mm, and a noise threshold of 0.04 mm. The initial scan mesh was then superimposed onto the post-scan mesh using the Gaussian best-fit method to align the surfaces and visualize the abrasion field on the post-mastication simulation scans. Volumetric loss (mm³) and vertical height loss (µm) were calculated by referencing the area of material loss with an integral function Tables 3, 4 and 5.

**Table 3:** Descriptive statistics (mean, standard deviation (SD), confidence interval (CI), Min/Med/Max and interquartile range (IQR)) for FL (N) of the different groups

Material	Treatment	Initial				Mastication simulator			
		Mean ± SD (*)	[95% CI]	Min/Med/Max	IQR	Mean ± SD (*)	[95% CI]	Min/Med/Max	IQR
VAR	PP1	1075 ±204^aAα^	[945;1205]	798/1015/1412	350	1350 ±226^aAβ^	[1207;1494]	901/1384/1638	389.4
	PP2	1331 ±290^abABα^	[1146;1515]	920/1232/1882	510	1299 ±337^abAα^	[1085;1514]	548/1374/1741	251.8
	PP3	1505 ±251^aBα^	[1345;1665]	1147/1488/1914	452	1192 ±328^aAβ^*	[983;1400]	522/1318/1605	467.9
	PP4	1257 ±182^aABα^	[1141;1373]	974/1260/1518	326	1267 ±330^aAα^	[1057;1477]	541/1239/1915	236.8
	PP5	1281 ±158^aABα^	[1181;1381]	982/1243/1555	199	1960 ±663^bBβ^	[1539;2382]	426/2157/2571	689.6
CRO	PP1	1073 ±144^aAα^	[982;1165]	870/1030/1330	239	1207 ±237^aAα^	[1056;1357]	818/1116/1546	400.5
	PP2	1273 ±427^aAα^	[1002;1544]	583/1234/2224	489	1059 ±377^aAα^	[819;1299]	497/1075/1537	715.3
	PP3	1295 ±498^aABα^	[979;1612]	516/1438/1962	926	887 ±486^aAα^	[579;1196]	259/989/1492	1002.04
	PP4	1255 ±394^aAα^	[1005;1505]	825/1121/2147	471	981 ±441^aAα^	[701;1261]	0/1106/1404	731.8
	PP5	1783 ±333^bBα^	[1572;1995]	1390/1677/2454	442	1327 ±474^aAβ^	[1026;1628]	563/1382/2053	751.3
FRE	PP1	1478 ±243^bABα^	[1323;1632]	1047/1535/1949	351	1436 ±579^aAα^*	[1069;1804]	387/1685/2155	945.6
	PP2	1652 ±175^bABCα^*	[1540;1763]	1473/1575/2026	261	1560 ±536^bAα^	[1219;1901]	578/1745/2162	994.5
	PP3	1910 ±237^bCα^	[1759;2060]	1491/1907/2262	319	1672 ±296^bAβ^	[1484;1860]	1199/1768/2097	533
	PP4	1282 ±309^aAα^	[1085;1478]	979/1188/2032	394	1399 ±532^aAα^	[1061;1737]	0/1521/2004	438.6
	PP5	1847 ±474^bBCα^	[1545;2148]	1009/1781/2539	857	1693 ±614^abAα^	[1302;2083]	591/1855/2379	1207.6

^abc^ indicate significant differences between materials within one post-polymerization protocol and one aging level

^ABCDE^ indicate significant differences between post-polymerization protocols within one material and one aging level

^αβ^ indicate significant differences between aging levels within one material and one post-polymerization protocol

*Deviation from the normal distribution

**Table 4:** Descriptive statistics (mean, standard deviation (SD), confidence interval (CI), Min/Med/Max and interquartile range (IQR)) for TBW (mm) of the different groups *n* = 12 per subgroup, except VAR with PP 3, CRO with PP 4 and FRE with PP 4 (*n* = 11); the two specimens with PP 4 had fractured during mastication simulation, and for one specimen of VAR with PP 3 no wear measurement is available

Material	Treatment	Mastication simulator			
		Mean ± SD (*)	[95% CI]	Min/Med/Max	IQR
VAR	PP1	-0.193±0.037^aA^	[-0.217; -0.170]	-0.250/-0.190/-0.130	0.065
	PP2	-0.188±0.036^aA^*	[-0.211; -0.165]	-0.250/-0.175/-0.150	0.068
	PP3	-0.210±0.039^aA^	[-0.236; -0.184]	-0.250/-0.220/-0.110	0.040
	PP4	-0.193±0.043^aA^	[-0.220; -0.165]	-0.240/-0.210/-0.110	0.065
	PP5	-0.180±0.037^aA^	[-0.203; -0.157]	-0.240/-0.180/-0.120	0.062
CRO	PP1	-0.352±0.414^aA^*	[-0.615; -0.089]	-1.580/-0.205/-0.160	0.055
	PP2	-0.293±0.378^aA^*	[-0.534; -0.053]	-1.480/-0.175/-0.100	0.118
	PP3	-0.215±0.103^aA^	[-0.280; -0.150]	-0.400/-0.180/-0.090	0.160
	PP4	-0.161±0.054^aA^	[-0.197; -0.125]	-0.270/-0.150/-0.100	0.070
	PP5	-0.182±0.103^aA^*	[-0.247; -0.116]	-0.470/-0.145/-0.110	0.070
FRE	PP1	-0.203±0.047^aA^	[-0.232; -0.173]	-0.280/-0.195/-0.140	0.077
	PP2	-0.257±0.103^aA^	[-0.322; -0.191]	-0.470/-0.235/-0.120	0.155
	PP3	-0.203±0.053^aA^	[-0.237; -0.169]	-0.310/-0.205/-0.130	0.080
	PP4	-0.227±0.106^aA^	[-0.299; -0.156]	-0.410/-0.190/-0.110	0.180
	PP5	-0.194±0.062^aA^	[-0.233; -0.155]	-0.360/-0.180/-0.130	0.047

^abc^ indicate significant differences between materials within one post-polymerization protocol

^ABCDE^ indicate significant differences between post-polymerization protocols within one material

*Deviation from the normal distribution

**Table 5:** Frequency distribution [%] for initial and artificial aged fracture types of the different groups

Material	Treatment	Fracture type initial				
		0 (No visible crack)	1 (One crack, with the crown remaining intact)	2 (Fracture into two fragments)	3 (Fracture into three fragments)	4 (Fracture into more than three fragments)
VAR	PP1	0 0[0;27]^V^	0 0[0;27]^V^	9 75[41;95]^W^	3 25[4;58]^V^	0 0[0;27]^V^
	PP2	0 0[0;27]^V^	0 0[0;27]^V^	5 41[14;73]^W^	6 50[20;79]^W^	1 8[0;39]^V^
	PP3	0 0[0;27]^V^	0 0[0;27]^V^	4 33[8;66]^WX^	1 8[0;39]^VW^	7 58[26;85]^X^
	PP4	0 0[0;27]^V^	0 0[0;27]^V^	10 83[50;98]^W^	2 16[1;49]^V^	0 0[0;27]^V^
	PP5	0 0[0;27]^V^	0 0[0;27]^V^	4 33[8;66]^WX^	5 41[14;73]^X^	3 25[4;58]^VWX^
CRO	PP1	0 0[0;27]^V^	0 0[0;27]^V^	6 50[20;79]^W^	6 50[20;79]^W^	0 0[0;27]^V^
	PP2	0 0[0;27]^V^	2 16[1;49]^V^	2 16[1;49]^V^	5 41[14;73]^W^	3 25[4;58]^WV^
	PP3	0 0[0;27]^V^	0 0[0;27]^V^	7 58[26;85]^W^	3 25[4;58]^V^	2 16[1;49]^V^
	PP4	0 0[0;27]^V^	2 16[1;49]^VW^	6 50[20;79]^X^	0 0[0;27]^V^	4 33[8;66]^WX^
	PP5	0 0[0;27]^V^	0 0[0;27]^V^	1 8[0;39]^V^	2 16[1;49]^V^	9 75[41;95]^W^
FRE	PP1	0 0[0;27]^V^	1 8[0;39]^V^	1 8[0;39]^V^	5 41[14;73]^W^	5 41[14;73]^W^
	PP2	0 0[0;27]^V^	0 0[0;27]^V^	2 16[1;49]^VW^	5 41[14;73]^W^	5 41[14;73]^W^
	PP3	0 0[0;27]^V^	0 0[0;27]^V^	1 8[0;39]^V^	2 16[1;49]^V^	9 75[41;95]^W^
	PP4	0 0[0;27]^V^	0 0[0;27]^V^	2 16[1;49]^VW^	6 50[20;79]^X^	4 33[8;66]^WX^
	PP5	0 0[0;27]^V^	0 0[0;27]^V^	0 0[0;27]^V^	2 16[1;49]^V^	10 83[50;98]^W^
Material	Treatment	Fracture type mastication simulator				
		0 (No visible crack)	1 (One crack, with the crown remaining intact)	2 (Fracture into two fragments)	3 (Fracture into three fragments)	4 (Fracture into more than three fragments)
VAR	PP1	0 0[0;27]^V^	0 0[0;27]^V^	8 66[33;91]^W^	4 33[8;66]^W^	0 0[0;27]^V^
	PP2	1 8[0;39]^V^	3 25[4;58]^VW^	6 50[20;79]^W^	2 16[1;49]^V^	0 0[0;27]^V^
	PP3	0 0[0;27]^V^	9 75[41;95]^W^	3 25[4;58]^V^	0 0[0;27]^V^	0 0[0;27]^V^
	PP4	0 0[0;27]^V^	0 0[0;27]^V^	9 75[41;95]^W^	2 16[1;49]^V^	1 8[0;39]^V^
	PP5	1 8[0;39]^VW^	1 8[0;39]^VW^	0 0[0;27]^V^	4 33[8;66]^WX^	6 50[20;79]^X^
CRO	PP1	0 0[0;27]^V^	0 0[0;27]^V^	9 75[41;95]^W^	2 16[1;49]^V^	1 8[0;39]^V^
	PP2	0 0[0;27]^V^	2 16[1;49]^V^	8 66[33;91]^W^	2 16[1;49]^V^	0 0[0;27]^V^
	PP3	0 0[0;27]^V^	4 33[8;66]^W^	3 25[4;58]^VW^	4 33[8;66]^W^	1 8[0;39]^VW^
	PP4	0 0[0;27]^V^	1 8[0;39]^V^	6 50[20;79]^W^	3 25[4;58]^VW^	1 8[0;39]^W^
	PP5	0 0[0;27]^V^	1 8[0;39]^VW^	4 33[8;66]^WX^	1 8[0;39]^VW^	6 50[20;79]^X^
FRE	PP1	0 0[0;27]^V^	0 0[0;27]^V^	4 33[8;66]^WX^	2 16[1;49]^VW^	6 50[20;79]^X^
	PP2	0 0[0;27]^V^	1 8[0;39]^V^	1 8[0;39]^VW^	4 33[8;66]^WX^	6 50[20;79]^X^
	PP3	0 0[0;27]^V^	1 8[0;39]^VW^	1 8[0;39]^VW^	4 33[8;66]^WX^	6 50[20;79]^X^
	PP4	0 0[0;27]^V^	0 0[0;27]^V^	0 0[0;27]^V^	5 41[14;73]^W^	6 50[20;79]^W^
	PP5	0 0[0;27]^V^	1 8[0;39]^V^	3 25[4;58]^V^	1 8[0;39]^V^	7 58[26;85]^W^

^VWXYZ^ indicate significant differences between fracture types within one material, one post-polymerization protocol and one aging level

### Fracture load measurement

FL was determined using a universal testing machine (Zwick 1445; ZwickRoell, Ulm, Germany) with a crosshead speed of 1 mm/min. The testing stamp with a diameter of 6 mm applied the load vertically on the occlusal surface in the central pit of the specimens. A tin foil (0.3 mm, Dentaurum, Ispringen, Germany) was positioned between the stamp and occlusal surface of the crowns to avoid loading peaks. The compatible software (testXpert V3.6; ZwickRoell) recorded the maximum load at the end of each measurement when fracture arose or when the load dropped by 10%.

### Fracture type analysis

**Fig. 2:**
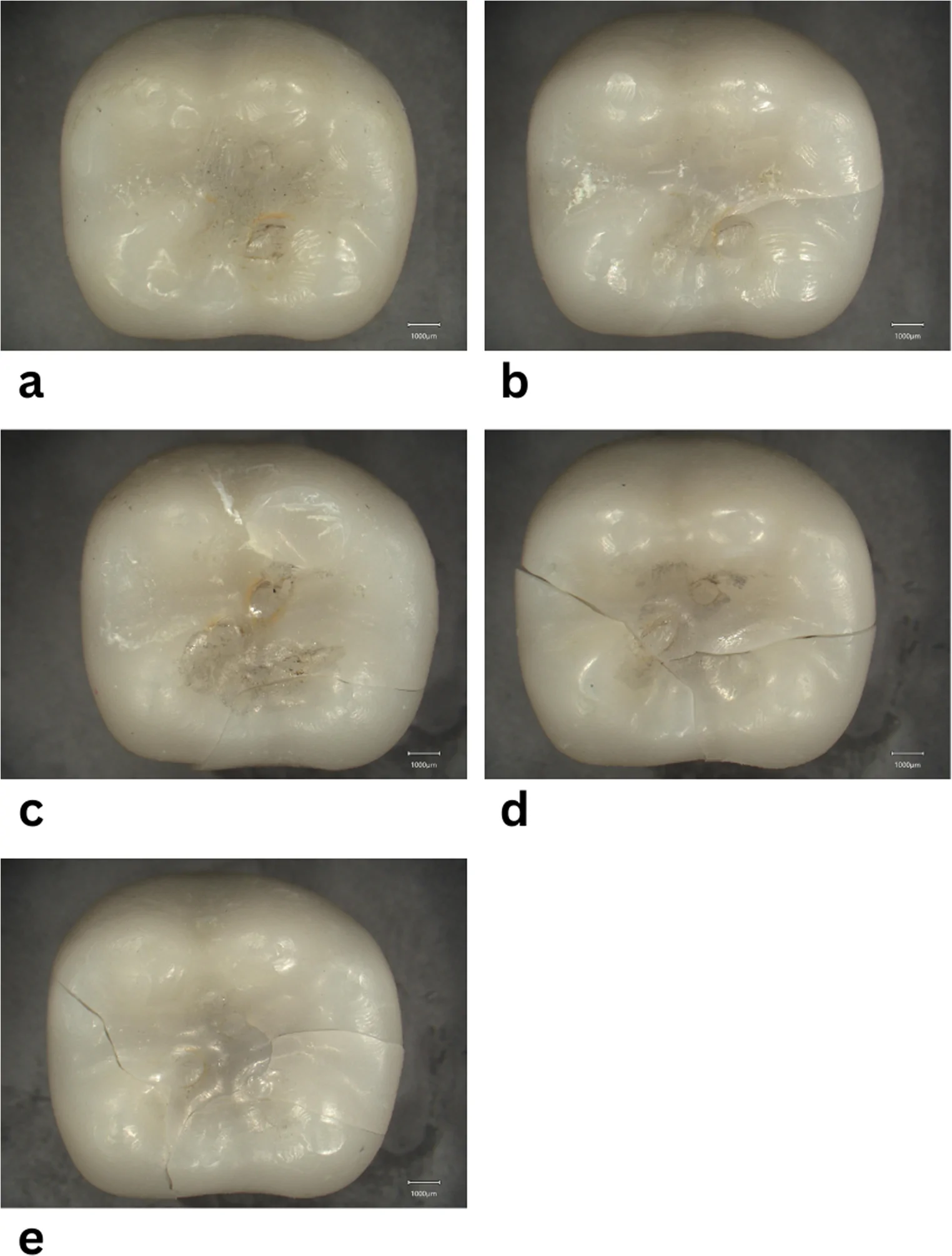
a-e: Fracture types during FL measurements: **a**: no visible crack (0), **b**: one crack with crown still intact (1), **c**: fracture into two fragments (2), **d**: fracture into three fragments (3) and **e**: fracture into more than three fragments (4)

Fractured specimens were examined using a digital light microscope (Keyence VHX-S600E; Keyence Corporation, Osaka, Japan) at magnifications of ×20 and ×50. Fracture types were classified as follows:

- No visible crack (0).
- One crack, with the crown remaining intact (1).
- Fracture into two fragments (2).
- Fracture into three fragments (3).
- Fracture into more than three fragments (4) (Fig. 2a–e).

### Statistical analysis

Data analysis was conducted using IBM SPSS Statistics Version 29.0 (IBM Corp., Armonk, NY, USA). Parametric tests were used for fracture load (FL) values because deviation from a normal distribution was observed in only 10% (3/30) of the cases. Normality was assessed with the one-sample Kolmogorov–Smirnov test with Lilliefors correction; the corresponding Shapiro–Wilk results are provided as Online Resource 1. Homogeneity of variances was assessed with Levene’s test, and the Welch-corrected t-test was used where the assumption was violated. Comparisons between the two aging levels were performed with independent-samples t-tests and are reported two-sided. A one-way analysis of variance (ANOVA) was performed to investigate significant differences among post-polymerization protocols (PPs) and materials. Fracture load was additionally analysed with a three-way analysis of variance (material × artificial aging × post-polymerization protocol) using Type III sums of squares, with partial eta squared (ηp²) as effect size and Scheffé’s test for post-hoc comparisons of the main effects. Since two specimens fractured during mastication simulation and were included with a fracture load of 0 N, this analysis was repeated excluding them (*N* = 358). The three-way analysis of variance constitutes the confirmatory analysis for fracture load; the one-way analyses within each material and aging level and the 15 pairwise comparisons between aging levels are reported as exploratory follow-up analyses of this model, without adjustment for multiple comparisons. Non-parametric tests were employed for two-body wear (TBW) measurements due to deviations from normal distribution in 26.67% (4/15) of the groups. The Kruskal-Wallis test was used to investigate significant differences among PPs and materials. Two-body wear was measured after mastication simulation only, so no initial-to-aged contrast is available for this outcome; data were available for 177 of 180 aged specimens. Statistical significance was set at *p* < 0.05. The distribution of fracture types was analysed with Pearson’s chi-square test on the cross-tabulation of the 30 material–aging–protocol groups against the five fracture-type categories (χ²(116) = 302.49, *p* < 0.001, *N* = 358). This was a single global test of the whole table; no pairwise comparisons between individual groups were performed, and no adjustment for multiple comparisons was therefore applied.

## Results

### Results for fracture load measurements

The full-factorial analysis of variance showed significant main effects of material (*p* < 0.001, ηp² = 0.152) and of the post-polymerization protocol (*p* < 0.001, ηp² = 0.133) on FL, whereas artificial aging had no significant main effect (*p* = 0.100, ηp² = 0.008). The interactions of material × aging (*p* = 0.001), aging × protocol (*p* = 0.011) and material × aging × protocol (*p* = 0.009) were significant, while the interaction of material × protocol was not (*p* = 0.082) (Table 6). The effect of the post-polymerization protocol on FL therefore depended jointly on the material and on the aging level. Across all materials and aging levels, PP 5 resulted in higher FL than all other protocols (Scheffé, all *p* ≤ 0.009), whereas PP 1 and PP 4 formed the lower end without differing from each other (*p* = 0.995).

**Table 6:** Full-factorial analysis of variance for fracture load (material × artificial aging × post-polymerization protocol; Type III sums of squares)

Source	Type III sum of squares	df	Mean square	F	*p*	ηp²
Corrected model	25 997 505.2	29	896 465.7	6.010	< 0.001	0.346
Material	8 817 299.7	2	4 408 649.8	29.555	< 0.001	0.152
Artificial aging	404 950.6	1	404 950.6	2.715	0.100	0.008
Post-polymerization protocol	7 545 193.2	4	1 886 298.3	12.646	< 0.001	0.133
Material × aging	2 038 778.4	2	1 019 389.2	6.834	0.001	0.040
Material × post-polymerization	2 111 959.7	8	263 995.0	1.770	0.082	0.041
Aging × post-polymerization	1 982 621.4	4	495 655.4	3.323	0.011	0.039
Material × aging × post-polymerization	3 096 702.2	8	387 087.8	2.595	0.009	0.059
Error	49 225 001.3	330	149 166.7			
Total	766 932 953	360				
Corrected total	75 222 506.5	359				

Primary analysis, *N* = 360, R² = 0.346 (adjusted R² = 0.288). Secondary analysis excluding the two specimens that fractured during mastication simulation (*N* = 358): material *p* < 0.001, artificial aging *p* = 0.185, post-polymerization protocol *p* < 0.001, material × aging *p* = 0.002, material × post-polymerization *p* = 0.094, aging × post-polymerization *p* = 0.005, material × aging × post-polymerization *p* = 0.003; corrected model *p* < 0.001, R² = 0.355. No term changed its significance status. Scheffé post-hoc comparisons of the main effect of the post-polymerization protocol: PP 5 > PP 1, PP 2, PP 3 and PP 4 (all *p* ≤ 0.009); no significant difference between PP 1 and PP 4 (*p* = 0.995)

#### Impact of post-polymerization

For the resin VAR, the highest initial FL was obtained with PP 3 (1505 N), followed by PP 2 (1331 N), PP 5 (1281 N) and PP 4 (1257 N); the lowest values were observed with PP 1 (1075 N) (one-way ANOVA F (4, 55) = 5.79, *p* < 0.001, η² = 0.296). Of these differences, only PP 3 versus PP 1 reached statistical significance (Scheffé, *p* < 0.001); the remaining protocols did not differ from either (Table 3). After artificial aging, PP 5 yielded the highest FL values compared to the other four PPs (*p* < 0.001). For the resin FRE, PP 3 produced the highest initial FL values (*p* < 0.001), whereas PP 4 resulted in the lowest values (*p* < 0.001). In contrast, the highest FL values for CRO were recorded with PP 5 (*p* < 0.001), while PP 1, PP 2, and PP 4 yielded the lowest values (*p* < 0.001). In CRO and FRE, artificial aging significantly reduced FL with PP 5 (1783 → 1327 N; *p* = 0.012) and with PP 3 (1910 → 1672 N; *p* = 0.041), respectively, whereas the remaining protocols were not significantly affected (Table 3).

#### Impact of materials

Following the application of PP 1, PP 2, and PP 3, the highest initial FL values were recorded for the resin FRE (*p* < 0.001, *p* = 0.012) (Table 3). With PP 5, VAR exhibited the lowest FL values compared to CRO and FRE (*p* < 0.001). Conversely, the highest FL values were observed for VAR after artificial aging and the application of PP 5 (*p* = 0.042). FRE achieved the highest values with PP 2 (*p* = 0.024) and PP 3 (*p* < 0.001). The application of PP 4 did not result in significant differences within initial or aged groups across all materials.

#### Impact of artificial aging

Artificial aging significantly increased FL in two of the 15 material–protocol combinations, both in resin VAR: with PP 1 (1075 → 1350 N; *p* = 0.005) and with PP 5 (1281 → 1960 N; *p* = 0.005). It significantly decreased FL in three combinations: VAR with PP 3 (1505 → 1192 N; *p* = 0.015), CRO with PP 5 (1783 → 1327 N; *p* = 0.012) and FRE with PP 3 (1910 → 1672 N; *p* = 0.041). In the remaining ten combinations the changes were not statistically significant, including CRO with PP 3 (1295 → 887 N; *p* = 0.055) (Table 3).

### Results for two-body wear measurements

Due to the significant correlation between height and volumetric loss values, all results are based on height-loss values (Table 4).

#### Impact of post-polymerization

No statistically significant differences in TBW values were observed among the five different PPs (Table 4). However, findings should be interpreted with caution due to the low statistical power.

#### Impact of materials

Similarly, no statistically significant differences in TBW values were found among the three resins (Table 4). Findings should also be interpreted with caution given the limited statistical power.

### Results for fracture types

Five fracture types were observed during FL measurements:

- No visible crack (0).
- One crack, with the crown remaining intact (1).
- Fracture into two fragments (2).
- Fracture into three fragments (3).
- Fracture into more than three fragments (4).

Two specimens fractured during mastication simulation at approximately 300,000 cycles, one from the CRO group and one from the FRE group, both post-polymerized with PP 4. Both were included in the fracture-load analysis with a value of 0 N, which is the conservative treatment of an early failure; a sensitivity analysis excluding them is reported in the footnote to Table 6. Because their occlusal surfaces were no longer intact, they could not be included in the two-body wear analysis, and they could not be classified for fracture type. The distribution of fracture types differed significantly across the 30 groups (Pearson chi-square, χ²(116) = 302.49, *p* < 0.001, *N* = 358). This single global test does not identify which groups differ; the following group-level observations are therefore descriptive. For resin VAR, fracture type 2 occurred most frequently with PP 1, PP 2, and PP 4, regardless of the aging regime (Table 5). PP 5 predominantly resulted in fracture type 3, followed by types 2 and 4, while PP 3 mostly resulted in fracture type 4In resin CRO, fracture type 2 was most often noted after the application of PP 3 and PP 4, and for PP 1, fracture types 2 and 3 occurred equally often compared to other fracture types. Increased occurrences of fracture type 4 were observed only with PP 5, regardless of the aging regime. After artificial aging, fracture type 2 was most frequent with PP 1, PP 2, or PP 4.

For resin FRE, fracture type 4 occurred significantly more often with PP 1, PP 3, and PP 5. PP 2 mostly resulted in fracture type 2, and PP 4 in fracture type 3. Within the artificially aged groups, fracture type 4 exhibited the greatest frequency among all tested PPs.

## Discussion

The effects of five different post-polymerization protocols on the FL and TBW of three 3D-printed resin materials for fixed dental prostheses were investigated in this study. The first null hypothesis was rejected for FL and retained for TBW: the post-polymerization protocol significantly affected FL but not TBW. The second null hypothesis was retained for the main effect of artificial aging on FL (*p* = 0.100) and for TBW, but the significant interactions of aging with material (*p* = 0.001) and with the protocol (*p* = 0.011) indicate that aging affected FL in a material- and protocol-dependent manner. The favourable performance of the two-capacitor flashlight protocol (PP 5) relative to the LED protocols may be related to its broader emission spectrum (280–700 nm) and to its higher irradiance [12], which together allow a wider range of initiator molecules to be activated. This interpretation does not extend to the one-capacitor protocol (PP 4), which formed the lower end of the fracture-load range together with PP 1, with no significant difference between the two (Scheffé, *p* = 0.995). That PP 5 (90 mW/cm²) outperformed PP 4 (37 mW/cm²) is consistent with previous studies demonstrating that higher light intensity of the photoinitiators specific wavelength results in more homolytic cleavages of photoinitiator molecules, thereby intensifying photopolymerization and increasing crosslink density [17, 18]. The broad-spectrum emission of flashlight devices may overcome the limitations of single-wavelength LED sources, which efficiently activate only photoinitiators with matching absorption profiles [14].

The photoinitiator system of the tested resins provides a plausible frame for the wavelength-dependent differences. According to the manufacturer’s information, VAR contains diphenyl(2,4,6-trimethylbenzoyl)phosphine oxide (TPO) as photoinitiator (Table 1). The absorption maximum of TPO lies in the near-UV range and its absorption extends into the violet region up to approximately 420 nm, whereas its absorption at 460 nm is low [19–21]. Initially, however, the highest FL values for VAR and FRE were obtained with PP 3 (460 nm), so the observed ranking is not explained by the absorption spectrum alone. The initiator system of CRO is not specified by the manufacturer, so no material-specific mechanism can be derived for this resin. In addition, the three LED protocols differed in irradiance as well as in wavelength [12]; wavelength and irradiance are therefore confounded, and no isolated wavelength effect can be derived from the present data.

Material- and aging-dependent responses to post-polymerization protocols were observed, reflecting differences in resin composition and filler content. VAR and CRO, designed for permanent applications, contain 30–50 wt% inorganic fillers, as stated by the manufacturer. Higher filler content did not, however, translate into higher fracture load in the present study: with PP 4 and PP 5, FRE reached initial values comparable to or higher than those of the two permanent resins (Table 3). Filler content alone therefore does not explain the observed material differences. Previous studies state that higher filler content correlates with improved wear behaviour in dental composites [7, 10]. However, in this study, no statistically significant influence on TBW could be demonstrated. When compared to CAD/CAM-milled materials, 3D-printed permanent resins exhibit lower biaxial flexural strength, with thermal aging negatively affecting performance more than in milled materials [4]. For provisional FDPs, additive manufacturing has been reported to provide better marginal and internal fit and higher fracture resistance than conventional methods [22, 23].

Artificial aging significantly reduced FL in three of the 15 material–protocol combinations — VAR with PP 3, CRO with PP 5 and FRE with PP 3 — which is consistent with known degradation mechanisms of polymer-based dental materials [24, 25], whereas FL increased significantly in two combinations and remained unchanged in the remaining ten. The combination of mechanical loading and thermal cycling in an aqueous environment leads to water absorption, hydrolytic degradation of the silane coupling agent at the filler-matrix interface, and accumulation of fatigue damage [26]. These findings align with TBW studies demonstrating that 3D-printed materials generally exhibit higher wear rates than CAD/CAM-milled composites, with printing orientation significantly influencing wear resistance: vertical printing (90°) has been shown to perform better than horizontal (0°) [27, 28]. Post-curing at a 405 nm wavelength, combined with temperatures exceeding 60 °C, has been shown to improve wear resistance, which provide both broad-spectrum light and thermal energy [29]. Since no significant differences in two-body wear were found between protocols in the present study, these reported mechanisms could not be confirmed here.

Interestingly, certain material-protocol combinations demonstrated increased FL values following artificial aging, particularly VAR with PP 1 and PP 5 (2/15 groups). This counterintuitive finding may potentially be attributed to continued polymerization phenomena, although the degree of conversion was not measured in this study to reinforce this hypothesis. During thermomechanical aging, elevated temperatures (55 °C) facilitate molecular mobility, enabling trapped free radicals to react with residual unreacted monomers, thereby increasing the degree of conversion and crosslink density [30]. Additionally, the thermal cycling may have induced stress relief within the polymer matrix, potentially reducing residual stresses from the printing process and resulting in more favorable mechanical behaviour. This hypothesis is supported by previous observations in CAD/CAM-milled polymeric prostheses, where aging over 7–21 days increased fracture load [31]. However, the material- and protocol-dependent nature of this effect suggests that the initial degree of conversion plays a critical role: materials with lower initial conversion may show improvement, while those with higher initial conversion undergo predominantly degradative processes. This interpretation is consistent with the observation that PP 5, despite its high initial energy input, still showed room for improvement through thermal post-curing effects. The exact mechanisms concerning the differential post-polymerization behaviour of PP 4 and PP 5 require further investigations, particularly regarding depth-dependent conversion profiles and residual monomer distribution following different flash-curing protocols.

Fracture type analysis provided additional insights into the failure mechanisms of 3D-printed dental restorations. The higher proportion of catastrophic failures (Type 4) in FRE specimens, suggests lower fracture toughness and energy absorption capacity compared to permanent resins. This is of great clinical relevance, as deformed crowns tend to lose occlusal contact and need to be replaced to prevent secondary caries or further damage of the tooth. PP 5 was also associated with an increase in catastrophic failures (Type 4), revealing that increased FL does not simultaneously translate into more favorable fracture types. Optimized post-polymerization increases absolute FL values but does not necessarily improve the material’s ability to resist crack propagation. Two specimens fractured during mastication simulation before FL measurements were performed, one from CRO and one from FRE group, both after the application of PP 4. Both specimens had been post-polymerized with PP 4, which was among the protocols with the lowest FL values in the present study (marginal mean 1240 N, not different from PP 1, *p* = 0.995); these early failures are therefore consistent with the fracture-load results.

The mean FL values observed in this study (887–1960 N) exceed reported maximum bite forces in the molar region (500–800 N), suggesting that properly post-polymerized 3D-printed restorations can withstand normal physiological loading [32]. However, the material- and protocol-specific effects of aging on mechanical properties underscore the importance of material selection and processing optimization for long-term applications. Masticatory forces range usually from 10 to 120 N, with maximum bite forces reaching 289–738 N in the premolar/molar region, though considerable individual variation exists [33]. The reference values cited above refer to children and adolescents up to 19 years of age [33]. At specimen level, the range extended down to 259 N in aged CRO with PP 3, and two specimens failed during mastication simulation, so a safety margin cannot be claimed for every specimen tested. The aging protocol of 480,000 cycles used in this study represents approximately 2 years of simulated clinical use.

Several limitations of this study should be acknowledged. The use of standardized crown geometries on metal abutments may not fully represent clinical conditions involving natural tooth preparations of varying geometries. While the artificial aging protocol is standardized and widely used, it cannot completely simulate the complex intraoral environment, including pH variations, enzymatic activity, and variable loading patterns. Additionally, the experimental post-polymerization devices used may not be directly comparable to commercially available equipment. The two specimens that fractured during mastication simulation were included in the fracture-load analysis with a value of 0 N, which lowers the mean of the PP 4 groups; a sensitivity analysis excluding them did not change the significance of any model term. Wavelength and irradiance were confounded across the three LED protocols, so no isolated wavelength effect can be derived. The degree of conversion was not measured, so the continued-polymerization interpretation remains a hypothesis. Future research should focus on exploring the findings of this in vitro study in clinical settings. All specimens were printed at a single orientation (0°), and each protocol used one fixed exposure (180 s per LED wavelength; 2,000 flashes for the flashlight device). Printing orientation and post-polymerization time have been shown to affect surface roughness, gloss, colour stability and Vickers hardness of additively manufactured materials [34, 35], so the protocols compared here cover only part of the relevant parameter space.

## Conclusions

Within the limitations of this in vitro study, the following conclusions can be drawn:

1. The FL of 3D-printed FDPs was significantly influenced by the post-polymerization protocol.
2. Favourable mechanical performance was observed with flashlight-based post-polymerization using two capacitors (PP5) compared to LED-based protocols across all tested materials under the experimental conditions given and materials that were tested.
3. Among the LED protocols, no single wavelength was favourable across all conditions: initially, 460 nm produced the highest FL for VAR and FRE, whereas 400 nm was intermediate. Because wavelength and irradiance were confounded across the LED protocols, these differences cannot be attributed to wavelength alone.
4. Artificial aging had no significant overall effect on fracture load. It significantly reduced FL in three and significantly increased it in two of the 15 material–protocol combinations, while the remaining ten were unaffected; the increases may reflect continued polymerization, which was not investigated in the present study.
5. The clinical success of 3D-printed dental restorations intended for long-term use is critically dependent on both material selection and optimization of the post-polymerization process.
6. No significant differences in two-body wear were found between protocols or materials; given the limited power of this comparison, small differences cannot be excluded.

## Data Availability

No datasets were generated or analysed during the current study.
